# 慢性淋巴细胞白血病免疫球蛋白重链可变区突变状态、基因片段使用特征及对预后的影响

**DOI:** 10.3760/cma.j.issn.0253-2727.2021.12.011

**Published:** 2021-12

**Authors:** 姮 李, 婷玉 王, 乐 尹, 培龙 王, 钊 程, 杰平 李, 薇 李, 飞跃 朱, 翠翠 吴, 自勉 罗, 艳华 陈, 红 肖, 世斌 邓, 永清 曹, 广森 张, 录贵 邱, 宏凌 彭

**Affiliations:** 1 中南大学湘雅二医院血液内科，长沙 410011 The Second Xiangya Hospital, Central South University, Changsha 410011, China; 2 中国医学科学院血液病医院（中国医学科学院血液学研究所），天津 300020 Institute of Hematology and Blood Disease Hospital, Chinese Academy of Medical Sciences (CAMS) & Peking Union Medical College (PUMC), Tianjin 300020, China; 3 南华大学附属长沙市中心医院，长沙 410000 Changsha Central Hospital Affiliated to Nanhua University, Changsha 410000, China; 4 湖南师范大学附属湖南省人民医院，长沙 410000 Hunan People's Hospital, The First Affiliated Hospital of Hunan Normal University, Changsha 410000, China; 5 娄底市中心医院，娄底 417000 Loudi Central Hospital, Loudi 417000, China; 6 岳阳市第二人民医院，岳阳 414000 The Second People's Hospital of Yueyang, Yueyang 414000, China; 7 湘潭市中心医院，湘潭 411100 Xiangtan Central Hospital, Xiangtan 411100, China; 8 南华大学附属第二医院，衡阳 421000 The Second Hospital, University of South China, Hengyang 421000, China; 9 湘潭市第一人民医院，湘潭 411100 The First People's Hospital of Xiangtan City, Xiangtan 411100, China; 10 永州市中心医院，永州 425000 Yongzhou Central Hospital, Yongzhou 425000, China; 11 长沙市第一医院，长沙410000 The First Hospital of Changsha, Changsha 410000, China

慢性淋巴细胞白血病（CLL）是西方国家成年人群中最常见的白血病类型，其特点是外周血成熟CD5阳性B淋巴细胞扩增[1]。具有不同遗传学特征的CLL患者的病程有明显异质性。

近年来，多种生物学指标被证实可影响CLL预后[2]。其中，免疫球蛋白重链可变区（IGHV）基因的突变状态是与CLL预后最为相关的预后因素之一[3]–[4]。通过荧光原位杂交（FISH）检测到的重现性遗传学异常，如染色体11q和17p缺失，已用于定义患者危险度分组[5]–[6]。但少有研究揭示IGHV基因突变状态与细胞遗传学之间的相关性[7]–[8]。中国CLL发病率低于西方国家，且发病年龄较小，侵袭程度更高，某些与预后相关的基因突变，如ATM、SF3B1、NOTCH1、MYD88 和TP53等，其发生率在东西方国家中存在差异[9]，提示中国CLL患者的疾病特征有可能与其独特的遗传学背景相关。本研究对多中心数据进行归纳总结，旨在进一步探索中国CLL患者IGHV基因突变特征及与预后的相关性。

## 病例与方法

1. 病例：回顾性分析湖南省及天津市多家中心的CLL患者资料。所有患者均签署知情同意书。本研究获中南大学湘雅二医院伦理委员会批准，伦理批号为（2021）伦审【临研】第（K067）号。诊断基于患者临床特征及实验室检查，符合国际CLL工作组指南所发布的诊断标准[10]。

2. 免疫表型：应用流式细胞术检测免疫表型，包括CD5，CD23、FMC7、CD22、CD25、CD19、CD20、CD10、CD11c及膜免疫球蛋白κ 轻链、λ轻链等抗原表达。

3. FISH检测：行骨髓FISH检测细胞遗传学异常，包括11q22.3（ATM）缺失、+12q15（MDM2）、13q14（RB1）缺失、17p13（TP53）缺失及CCND1/IGH易位。10例具有正常核型和非血液系统恶性疾病的患者作为正常对照。阈值定义为正常对照的平均值+3×标准差，各探针阳性阈值如下：+12：7.5％，TP53缺失：5.0％，RB1 或ATM缺失：6.5％，IgH易位：4.5％。

4. IGHV突变状态检测：应用多重PCR法检测CLL患者IGHV片段使用情况及突变状态[11]–[12]。将免疫球蛋白重链（IgH）序列同源性<98％定义为体细胞突变，而IgH序列同源性≥98％定义为未发生体细胞突变[13]。

5. 随访：通过查阅患者电子病历及纸质病历确认患者住院治疗情况，对患者进行电话随访，随访时间截至2020年3月。治疗间隔时间（TTFT）定义为确诊之日起至达治疗指征开始首次治疗、死亡或末次随访（尚未达到治疗指征患者）的时间。

6. 统计学处理：采用SPSS 19.0软件进行统计学分析。计数资料的组间比较应用*χ*^2^检验或Fisher精确检验，计量资料的组间比较采用Mann-Whitney非参数检验。采用Kaplan-Meier法及Log-rank检验计算预期TTFT并绘制生存曲线。*P*<0.05为差异有统计学意义。

## 结果

1. 临床特征：287例患者中，男204例，女83例。中位年龄为58（26～86）岁。伴有淋巴结肿大的患者占74.2％，伴肝、脾肿大的患者分别占6.4％和43.5％。中位淋巴细胞绝对计数为21.75（0.6～367.61）×10^9^/L，中位HGB水平为126（36～177）g/L，中位PLT为148（9～370）×10^9^/L。26％的患者乳酸脱氢酶（LDH）水平升高，87.6％的患者伴β_2_-微球蛋白（β_2_-MG）水平升高。在行FISH及基因突变检测的232例患者中，最常见的遗传学异常为染色体13q缺失（23.3％），其后依次为12号染色体三体（22.9％）、染色体17p缺失/TP53基因突变（15.8％）、染色体11q缺失（14.9％）、SF3B1突变（7.7％）、MYD88L265P突变（7.6％）及BIRC3突变（5.3％）。初诊时仅有14.6％的患者处于Rai 0期，其余患者均处于进展期，Ⅰ、Ⅱ、Ⅲ、Ⅳ期占比分别为31.9％、21.7％、12.6％和19.3％。

2. IGHV突变状态及VH基因使用情况：287例患者中，IGHV基因为突变状态者占66.2％（190/287），突变中位值为5.6％（0～22.0％）。7个VH基因家族中最常见的VH基因家族为VH3（44.0％）, 其后依次为VH4（30.5％）、VH1（17.7％）、VH2（3.2％）、VH5（1.8％）、VH6（1.8％）、VH7（1.1％）。使用频率在5％以上的VH基因片段包括VH4-34（11.7％）、VH4-39（8.5％）、VH3-30（8.2％）、VH3-23（6.7％）、VH1-69（6.0％）及VH3-70（5.3％）。使用VH3-21基因片段的患者占2.8％。7个基因家族中IGHV基因突变率呈不均一分布（图1A）。在使用VH3基因家族的患者中，78.2％的患者发生体细胞突变，突变率显著高于其他基因家族（*P*<0.001）。而在使用VH1基因家族的患者中，突变率仅为36％，显著低于其他基因家族（*P*<0.001）。进一步分析常见VH基因片段中的突变情况，突变率最高的基因片段为VH4-34（90.9％），显著高于其他基因片段（*P*＝0.001）。VH4-39和VH1-69片段的突变率分别为41.7％和17.6％，显著低于其他基因片段（*P*＝0.009、*P*<0.001）（图1B）。

**图1:**
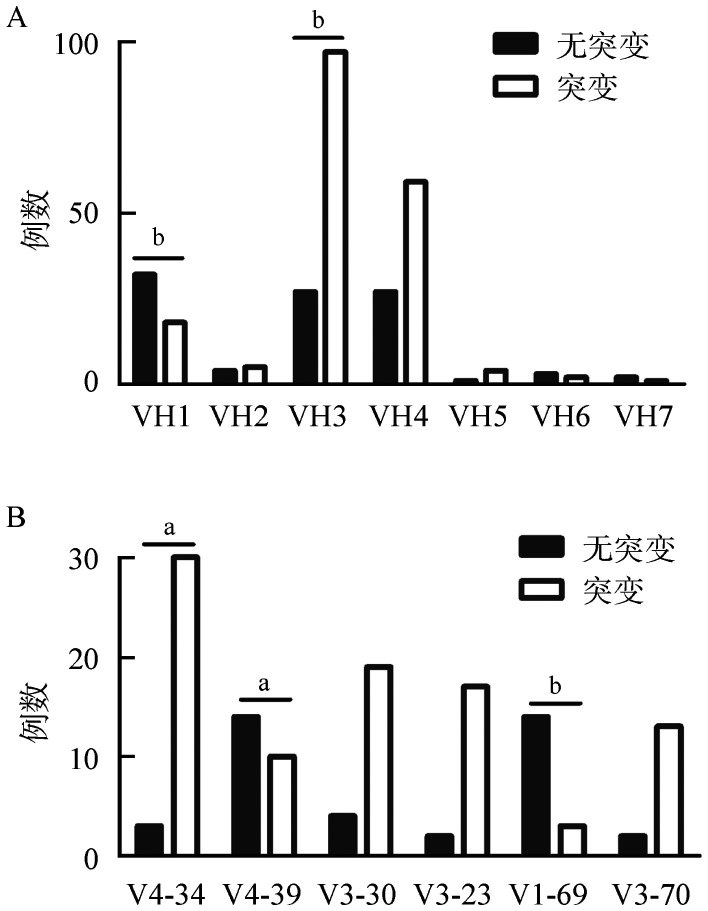
VH基因家族各成员的突变率分布 A：使用不同VH基因家族的慢性淋巴细胞白血病（CLL）患者中IGHV基因突变情况；B：使用不同VH基因片段的CLL患者中IGHV基因突变情况。^a^ *P*<0.01；^b^ *P*<0.001

3. IGHV突变状态与临床特征相关性：将患者按照IGHV基因有无突变分为两组，两组间临床特征的比较见表1。IGHV为突变状态的患者相较于IGHV无突变者合并染色体13q缺失的比例更高（28.2％对13.2％，*P*＝0.015）。而TP53基因缺失或突变比例在IGHV无突变组更常见（*P*<0.001）。IGHV无突变组患者中有28.6％伴有TP53基因缺失或突变，而突变组中仅9.6％的患者伴有TP53基因缺失或突变。两组患者的中位年龄、性别比、临床分期、血细胞计数、β_2_-MG水平、LDH水平之间差异均无统计学意义（*P*值均>0.05）。另外，使用VH4-34片段的患者中均不伴有TP53基因缺失或突变，显著低于使用其他基因片段的患者（0 对 15.8％，*P*＝0.026）。

**表1:** 免疫球蛋白重链可变区（IGHV）基因有、无突变的CLL患者临床特征比较

特征	M-CLL（190例）	UM-CLL（97例）	*χ*^2^值或*t*值	*P*值
年龄［岁，*M*（范围）］	58（36～86）	59（26～80）	0.330	0.658
性别（例，男/女）	138/52	66/31	0.658	0.417
Rai 分期（％）			0.919	0.632
低危	17.4	12.7		
中危	53.9	54.9		
高危	28.7	32.4		
淋巴结肿大（％）	70.1	82.1	2.790	0.095
脾大（％）	43.3	43.9	0.008	0.927
肝大（％）	5.0	9.2	1.306	0.253
淋巴细胞计数［×10^9^/L，*M*（范围）］	22.56（0.64～367.61）	16.42（0.66～252.7）	0.073	0.942
HGB［g/L，*M*（范围）］	126.5（36～177）	125.5（46～175）	0.321	0.749
PLT［×10^9^/L，*M*（范围）］	148（9～370）	150（10～349）	−1.085	0.280
β_2_-MG升高（％）	87.9	86.8	0.027	0.869
LDH升高（％）	24.1	31.0	1.018	0.313
遗传学异常（％）				
13q−	28.2	13.2	5.875	0.015
+12	22.0	26.4	0.384	0.535
11q−	13.8	19.4	1.019	0.313
17p−/TP53突变	9.6	28.6	13.266	<0.001

注：CLL：慢性淋巴细胞白血病；M-CLL：伴IGHV基因突变CLL；UM-CLL：无IGHV基因突变CLL；β_2_-MG：β_2_-微球蛋白；LDH：乳酸脱氢酶

4. IGHV对TTFT的影响：中位随访时间为21个月，234例患者中有159例达到治疗指征并启动治疗。Rai低危组（Rai 0期）患者中位TTFT为36.0个月，而中危组（Rai Ⅰ～Ⅱ期）和高危组（Rai Ⅲ～Ⅳ期）患者的中位TTFT分别为20.0个月和12.0个月（*P*＝0.020）。同时，IGHV无突变（16.0个月对36.0个月，*P*＝0.001）、TP53基因异常（19.0个月对25.0个月，*P*＝0.025）和LDH水平升高（12.0个月对25.0个月，*P*＝0.017）也是显著影响TTFT的预后不良因素。MYD88基因突变和 SF3B1基因突变虽然在CLL患者中较常见，但对TTFT无显著影响。

进一步分析IGHV突变状态在不同临床分期亚组患者中的预后意义，在Rai低危组患者中，IGHV基因无突变者较突变者TTFT显著缩短［35.0（95％ *CI* 15.6~54.4）个月对103.2（95％ *CI* 76.7~129.7）个月，*P*＝0.002］（图2A）。然而在Rai中高危组患者中，IGHV基因突变状态则对TTFT无显著影响［32.8（95％ *CI* 22.6~43.1）个月对62.4（95％ *CI* 46.3~78.5）个月，*P*＝0.102］（图2B）。

**图2:**
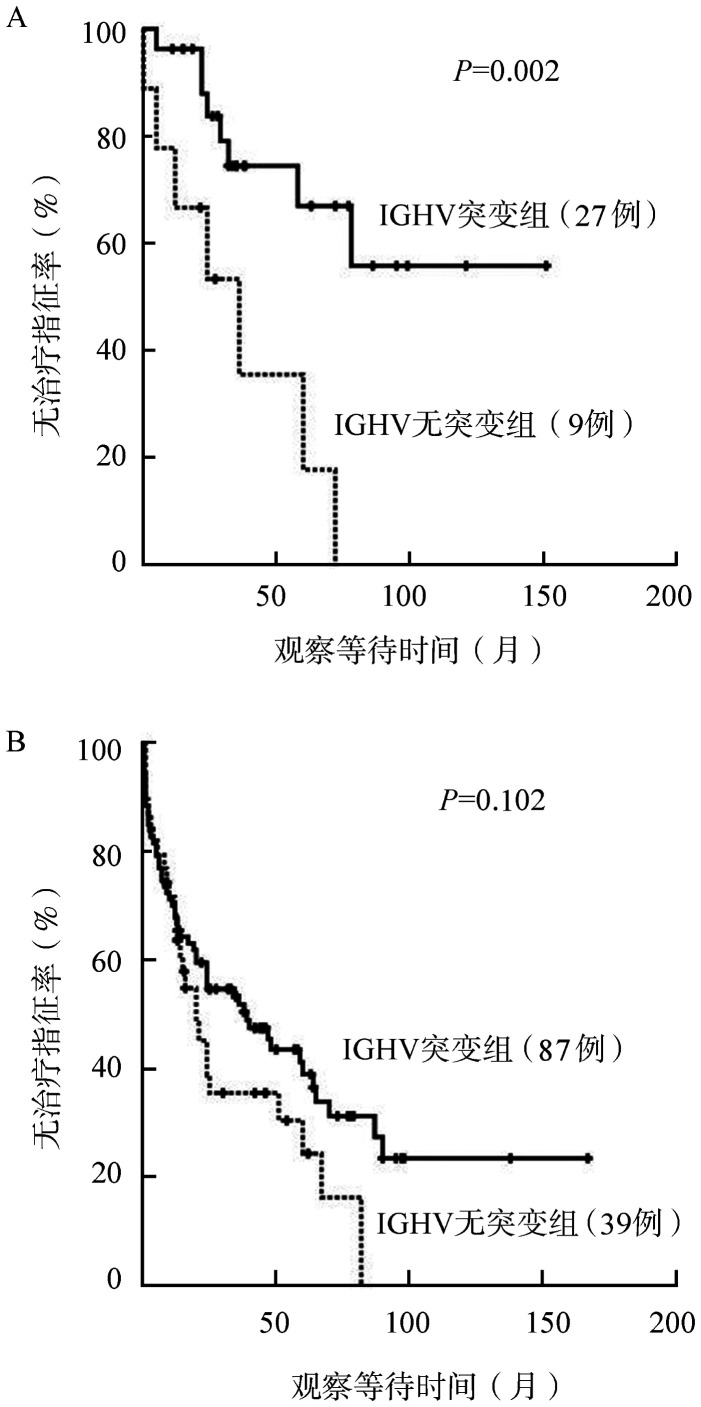
IGHV突变状态对慢性淋巴细胞白血病（CLL）不同危险度患者治疗间隔时间（TTFT）的影响 A：Rai危险度分层为低危组的CLL患者中IGHV基因突变状态对TTFT的影响；B：Rai危险度分层为中高危组的CLL患者中IGHV基因突变状态对TTFT的影响

## 讨论

有不少研究探讨了IGHV基因突变状态对CLL患者的预后的影响，但不同种族的CLL患者使用的VH基因片段存在差异。对于亚洲CLL人群的IGHV基因使用情况及对预后的影响还需要更深入的研究。在西方人群中，最常见的基因家族为VH1、VH3和VH4[14]–[17]。本研究中，VH3基因家族使用比例更高（44.0％），其次为VH4基因家族（30.5％）和VH1基因家族（17.7％），其余基因家族使用率较低，这与中国的另一研究相近[18]。VH1基因家族的使用率显著低于西方CLL人群（和美国[17]相比，*P*<0.001；和意大利[19]相比，*P*＝0.030）。

VH基因片段的使用情况在东西方CLL人群中也存在差异。VH1-69是在西方CLL人群中最常见的基因片段，在美国CLL人群中为11％～18％[17],[20]，在欧洲CLL人群中则为10.7％～11.4％[16],[21]–[22]，而在本研究中表达VH1-69的患者仅为6.0％。表达VH4-34、VH4-39、VH3-30及VH3-23的患者总和占本组患者的35％，而在英国及斯堪的纳维亚CLL人群中，这些基因片段的表达比例总和仅为约20％[16],[22]–[23]。VH4-34为本组患者中最常见的基因片段，与其他东亚CLL人群的研究一致[9],[24]，但在中东则以VH3-7基因片段最为常见[25]。

在西方CLL人群中，IGHV突变率约为50％[26]，本研究中IGHV基因突变比例更高（66.0％），与其他亚洲CLL的研究相近[18],[27]。突变率最高的基因家族为VH3和VH4基因家族，与国外报道一致[17],[28]。表达VH4-34基因片段的患者体细胞突变比例更高[16],[27]，提示患者预后更好。本研究中，表达VH4-34基因片段的患者体细胞突变比例高达90.9％，同时，无患者伴有染色体17p缺失或TP53基因突变。由于染色体17p缺失或TP53基因突变在IGHV基因无突变的CLL患者中更常见，我们推测这可能是表达VH4-34基因片段的患者中出现TP53基因异常的比例偏低的原因之一。

与VH4-34基因片段不同，大部分表达VH4-39和VH1-69基因片段的患者体细胞突变比例较低。以往认为，在亚洲CLL人群中，VH1-69基因片段的使用比例很低[18],[24]，但在本研究中，VH1-69的使用比例排在第5位。VH1-69的表达被认为与不良预后相关，在东西方人群中均有报道[29]–[30]。本研究中VH1-69的使用比例显著低于瑞典[22]（*P*<0.001）和美国[17]（*P*＝0.040），而与其他国家的研究相比则差异无统计学意义。VH4-39基因片段发生体细胞突变比例偏低（41.7％），提示预后更差，本研究中VH4-39基因片段使用比例显著高于国外研究（和英国[16]相比，*P*＝0.044；和意大利[19]相比，*P*＝0.002）。

VH3基因家族中的多数基因片段突变比例较高，如VH3-30、VH3-23和VH3-7的突变率均超过80％。其他研究也曾报道VH3-7基因片段常见于IGHV基因有突变的CLL患者[16]–[17],[23]。虽然VH3-21基因片段突变比例也较高，但有研究提示表达VH3-21基因片段者无论突变与否，均预后不良[31]。本研究中VH3-21基因片段的使用率与多数研究相近，但显著低于日本[24]和瑞典CLL人群[22]。

IGHV基因突变状态在CLL患者中的预后价值已非常明确，即伴IGHV突变的患者预后优于无突变者[2],[32]。本研究中IGHV突变状态仅在Rai低危组患者中显著影响TTFT，而在Rai中高危患者组中，IGHV突变状态则对TTFT无显著影响。表明进展期患者开始启动治疗的间隔时间不受IGHV突变状态的影响。

IGHV基因片段重排在不同地域和不同人种之间存在差异，即使在亚洲人群中，VH基因片段的使用情况也不尽相同，其潜在机制也许和IGHV胚系基因构成以及环境因素相关[21]。不同人群之间除表达不同的IGHV基因片段之外，互补决定区3（CDR3）的序列也存在差异，提示病原体或自身抗原在CLL的发病过程中扮演重要角色[33]，遗憾的是由于本研究中患者CDR3序列信息不完善而无法分析。综上，本研究进一步揭示了中国CLL人群和西方CLL人群之间IGHV片段的表达差异，为CLL起源和发病机制提供更多理论依据。
